# News and Miscellany

**Published:** 1902-10

**Authors:** 


### News and Miscellany.

---

**A Good Cash Practice** can be had by buying a physician's property fifteen miles from San Antonio on railroad, German neighborhood. Reply to G., "Red Back" (*Texas Medical Journal*), Austin, Texas.

---

**Samuel Van Dyke Stout,** youngest son of the venerable ex-Medical Director of Confederate Hospitals, Dr. Samuel Hollingsworth Stout now of Dallas, died at his home in Atlanta, Ga., August 29, 1902.

---

**Dr. P. M. Payne,** an accomplished specialist in diseases of the eye, ear, nose and throat, and late oculist to the American Hospital in the City of Mexico, and to the Mexican National Railroad, has located at Brownwood, Texas. I commend him to my friends and the profession at large as an estimable gentleman, an ethical physician, and an experienced and skillful oculist and aurist.

Dr. Payne was a pupil of the great DeRoaldes of New Orleans, and for two years was resident physician at the New Orleans Eye, Ear, Nose and Throat Hospital, under Dr. DeRoaldes. See his card in advertising pages.

---

**The State Board of Homeopathic Examiners** will meet in Dallas, October 7 and 8, inst., and at the same time the State Homeopathic Medical Society will meet. Big day in Dallas—Southwestern Tri-State Medical Association in session there same day, and State Fair going on all the while.

---

**Dr. T. C. Osborn,** an account of whose death appeared in our last issue, was born in Nashville, and not in Rutherford county, Tennessee, as stated in the Cleburne papers. I reproduced the biographical data from the Cleburne papers, and supposed of course that it was correct as it must have been furnished by the family. Dr. Stout of Dallas, a life long friend of Dr. Osborn, called my attention to the error.

---

**New Orleans Polyclinic.**—Sixteenth annual session opens November 3, 1902, and closes May 30, 1903. Physicians will find the Polyclinic an excellent means for posting themselves upon modern progress in all branches of medicine and surgery. The specialities are fully taught, including laboratory work. For further information address NEW ORLEANS POLYCLINIC, Postoffice Box 797, New Orleans, La.

---

**I want** the readers of the "Red Back" to see Dr. Church's card, elsewhere in this issue. Dr. Church at one time was Assistant Superintendent of the North Texas Insane Asylum. He is now at the head of the Eye, Ear, Nose and Throat Hospital in Los Angeles, California, and is one of the leading men of California. Daniel of the "Red Back" endorses him right straight along —all the way through.

---

**Meeting of the Board of Medical Examiners for the State of Texas.**—The next meeting of the Board of Medical Examiners for the State of Texas will be held in Dallas, Texas, beginning October 7, 1902, to examine applicants who desire to practice medicine in Texas, and to transact any other business that may come up before the Board. All applicants who desire to take said examinations must be present on the morning of the first day of meeting.

For application blanks, copy of law, rules governing examination and any other information, address the secretary.

M. M. Smith, M. D., Secretary,

Austin, Texas.

J. T. Wilson, M. D., President,

Sherman, Texas.

